# The Effect of an Informative Video upon Anxiety and Stress in Patients Requiring an Oral Biopsy: A Randomized Controlled Study

**DOI:** 10.3390/ijerph19020783

**Published:** 2022-01-11

**Authors:** Oscar Toralla, Pia Lopez Jornet, Eduardo Pons-Fuster

**Affiliations:** 1 Departamento Odontologia, Universidad San Carlos de Guatemala, Guatemala 01018, Guatemala; otoralla@gmail.com; 2 Faculty of Medicine and Odontology, University of Murcia, 30008 Murcia, Spain; eduardo.p.f@um.es

**Keywords:** oral biopsy, dental anxiety, video, state-trait anxiety inventory

## Abstract

Objective: The use of multimedia tools improves patient understanding of surgical procedures, reduces anxiety and increases satisfaction. The present study evaluates the impact of an audiovisual intervention (video) upon anxiety and stress in patients requiring an oral biopsy. Material and methods: A prospective randomized clinical trial was carried out in patients requiring an oral biopsy. The control group (*n* = 60) received verbal standard information while the experimental group (*n* = 60) received information in the form of a video. The following data were recorded: gender, age, educational level and hemodynamic parameters (blood pressure, heart rate and blood oxygen saturation). The following questionnaires were used to assess anxiety and stress before and after the biopsy procedure: Corah’s Modified Dental Anxiety Scale (MDAS), the State-Trait Anxiety Inventory (STAI) and the Hospital Anxiety and Depression Scale (HADS). Results: The final study sample consisted of 120 patients, of which 65.8% were women and 34.2% men, with a mean age of 40.5 ± 15.3 years. At the end of the study, the experimental group presented a significantly lower MDAS score than the control group (*p* = 0.041). The STAI score also showed a significant decrease with respect to the control group at the end of the study (*p* = 0.012). There were no statistically significant changes in the hemodynamic parameters in either group. Conclusions: The video constituted a useful and easy tool for reducing anxiety among patients requiring an oral biopsy.

## 1. Introduction

The clinical identification and evaluation of potentially malignant oral lesions can allow the detection of up to 99% of all oral cancers [1,2]. As recommended by the World Health Organization (WHO), any suspicious oral lesion that fails to resolve in two weeks after identification and the elimination of local causal factors should be subjected to biopsy [2,3,4,5]. Surgical biopsy remains the gold standard for the diagnosis of oral cancer [1,2,3]. Early detection and screening have been shown to be effective in reducing morbidity and mortality associated to oral malignancies [2,3,4,5,6,7]. At present, most oral cancers are diagnosed in advanced stages of the disease, with the application of invasive treatments, and the survival rates remain poor [2].

In the year 2000, Hägglin et al. conducted a literature review in women and found the prevalence of dental anxiety and fear of the dentist to range between 6–20% [8]. This situation contributes to poor dental care and, most importantly, to delays in diagnosis [3,4,5]. Oral biopsy is an invasive surgical procedure that causes stress in both patients and people close to them [9,10]. Specifically, oral biopsy tends to generate anxiety before the actual procedure is carried out, due to wrong ideas and misconceptions, fear of injury and concerns about the disease that can be diagnosed as a result of the biopsy [11]. The management of stress and anxiety is not always easy.

It has been reported that women experience more anxiety than men [11], and that it is more frequent in young adults [12]. Some studies have related anxiety to a low educational level [13], while others have recorded greater anxiety among individuals with a higher educational level [14]. Although oral biopsy is a simple technique that usually causes only mild injury, there are a number of adverse effects that must be taken into account. In clinical practice, patients scheduled for biopsy often suffer substantial stress before, during or after the procedure [15,16,17]. In particular, most patients feel uncomfortable both with the procedure itself and with the idea of the results of the biopsy.

Providing adequate information is crucial, though there is no agreement as to which is the best way to present such information. Although verbal information provided by the professional is the most common scenario, the use of written information in the form of explanatory leaflets, as well as audio recordings or videos, has also been proposed [18,19,20,21,22,23,24,25,26].

The present study was carried out at the Dental School of Universidad de San Carlos (Guatemala) to evaluate the hypothesis that an audiovisual intervention (video) providing information on oral biopsy is able to reduce patient anxiety and stress.

## 2. Materials and Methods

A prospective, randomized clinical trial was carried out involving 165 initially eligible patients, of which 45 failed to meet the inclusion criteria. A total of 120 patients were therefore finally randomized, following the Consort Statement guidelines, with the definition of 2 groups: a control group (*n* = 60) that received standard verbal information, and an experimental group (*n* = 60) that received information in the form of a video. The study was approved by the Ethics Committee of Universidad de San Carlos (Guatemala) (REF adcobiusac 023-2021) (NCT05164068) and was carried out in abidance with the principles of the Declaration of Helsinki. All patients gave written informed consent to participation in the study.

**Figure 1:**
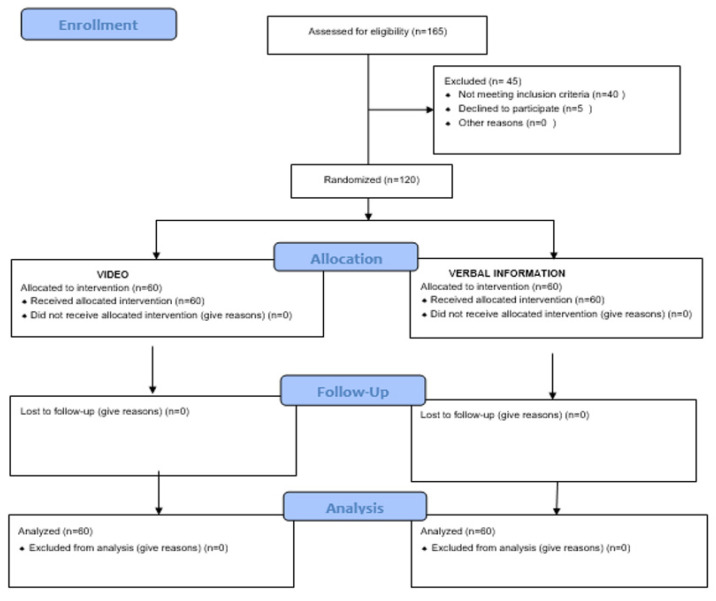
Consort 2010 flow diagram.

The patients were referred to the Dr. Cesar Lopez Acevedo histopathology and multidisciplinary laboratory of the clinics at the Dental School of Universidad de San Carlos, for the diagnosis of lesions of the oral cavity requiring an oral biopsy (Figure 1).

The inclusion criteria were a patient age of over 18 years and the presence of an oral lesion/s requiring biopsy. Patients with decompensated systemic disorders, poor general condition, a history of psychiatric disease or psychoactive drug use were excluded, as were pregnant women and individuals failing to sign the informed consent document.

The patients were randomized to the two study groups using a software application (www.randomization.com, accessed on 10 October 2021). The experimental group likewise consisted of 60 individuals that received information about the biopsy procedure in video format. The video was developed by the investigator (OT) for research purposes and had a duration of 2 min. The video contents were clear and adapted to the characteristics of the population of Guatemala. The video provided a simple description of the biopsy, its risks and benefits, complications and general recommendations, and was presented preoperatively using an electronic tablet and wireless earphones, in a room with a relaxed environment. The control group consisted of 60 patients that received face-to-face verbal information in a homogeneous and reproducible manner (the duration and content of the biopsy information was similar to the video).

Hemodynamic parameters (systolic and diastolic blood pressure, heart rate and oximetry) were recorded, (initial and final) and the following questionnaires were administered:

Corah’s Modified Dental Anxiety Scale (MDAS) is a questionnaire designed specifically to measure anticipatory fear and anxiety. It comprises 5 questions with multiple choice single-selection responses, whereby the subject chooses the response closest to his or her feelings. Scores range from 5 (no anxiety) to 25 (maximum anxiety). The lower limit for defining subjects with extreme anxiety is 19 [27].

The State-Trait Anxiety Inventory (STAI) is composed of 40 items, of which 20 evaluate anxiety state (representing a transient emotional situation, i.e., intensity of anxiety assessed at a point in time) and the other 20 evaluate anxiety trait (representing the tendency towards anxiety, i.e., assessed in terms of frequency). The instrument is scored from 0–4 (0 = not at all/almost never/; 1 = a little/sometimes; 2 = quite a lot/often; and 3 = a lot/almost always) [28].

The Hospital Anxiety and Depression Scale (HADS) was used to analyze the psychological profile of the patients. This instrument consists of two subscales related to anxiety and depression. Each subscale comprises 7 items that assess disorders of mood state. A score of over 10 is indicative of probable anxiety or depression, while scores of ≤7 are indicative of no significant anxiety or depression [29].

### 2.1. Oral Biopsy

A clinical evaluation of the oral cavity was carried out. The biopsies were all performed under local anesthesia with a vasoconstrictor by the same surgeon (OT) who was blinded to the group to which the patient belonged. The total duration of the biopsy procedure was recorded in each case. All patients received postoperative instructions, with the prescription of a nonsteroidal anti-inflammatory drug (ibuprofen during 4 days) and a 0.2% chlorhexidine rinse.

### 2.2. Sample Size

The study sample consisted of 120 individuals (60 per group), and thus the margin of error was 4.5% for a level of confidence of 95%, with the assumption of maximum variance (σ2 = 0.25).

All the questionnaires were administered and the hemodynamic parameters (blood pressure, heart rate and oximetry) were recorded before and immediately after the oral biopsy.

### 2.3. Statistical Analysis

The statistical analysis was performed using the SPSS version 25.0 statistical package (IBM Corporation, Armonk, NY, USA) for MS Windows. Statistical significance was considered for *p* < 0.05.

Categorical variables were reported as absolute frequencies and percentages, while continuous variables were reported as the mean and standard deviation (SD) and minimum and maximum value. Qualitative parameters were compared between groups based on the chi-squared test, while quantitative parameters were compared using the Student *t*-test, following the confirmation of normal data distribution with the Kolmogorov–Smirnov test and of the homogeneity of variances with the Levene test.

In order to determine whether the changes in the scales over time were dependent on the visualization of the informative video or not, we used the two-factor analysis of variance (ANOVA) with repeated measures in one of them, based on the general linear model (GLM), to study the impact of intra- (time: pre-post measures) and inter-subject factors (group: no visualization versus visualization of the video) upon the dependent variables (hemodynamic parameters and questionnaire scores), and their interactions (Group*Time).

## 3. Results

The final study sample consisted of 120 patients (50% in the control group and 50% in the experimental group), of which 65.8% were women and 34.2% men, with a mean age of 40.5 ± 15.3 years. Table 1 describes the demographic parameters and habits of the patients considered globally and by groups. No statistically significant differences were observed between the experimental group and the control group in terms of age or gender, smoking or alcohol intake, though there were significant differences in tooth brushing frequency. In relation to the oral biopsy, a significant difference in the duration of the surgical procedure was observed (Table 2). Most of the biopsy results were exophytic lesions (papilloma; oral fibromas).

Table 3 shows the hemodynamic parameters corresponding to all the patients of the study, as well as the comparisons between those individuals that visualized the video (experimental group) and those who did not (control group).

With regard to the impact of visualization of the video, a two-factor ANOVA was performed to assess the effect of the intra- (time: pre-post measures) and inter-subject factors (group: no visualization versus visualization of the video) upon the dependent variables (hemodynamic parameters and questionnaire scores), and their interactions (Group*Time). Table 4 shows the results corresponding to systolic and diastolic blood pressure, heart rate and blood oxygen saturation (oximetry). There were no statistically significant differences in the values at the end of the biopsy procedure versus baseline between those individuals that visualized the video (experimental group) and those who did not (control group).

Table 5, in turn, reports the results corresponding to the different scales employed. The MDAS scores evidenced significant reductions over time, independently of the group involved (8.14 versus 7.80; *p* = 0.021). However, a significant effect of the group and time interaction was recorded. Specifically, in the control group, no significant difference was observed in the MDAS score between baseline and the end of the biopsy procedure (*p* = 0.754), while in the experimental group the score at the end of the biopsy procedure was significantly lower than at baseline (*p* = 0.005). At the end of the operation, the patients in the experimental group showed a significantly lower MDAS score than the patients in the control group (*p* = 0.041).

In relation to anxiety state (STAI), the results evidenced a significant decrease in the score over time, independently of the group involved (31.09 versus 30.0; *p* = 0.002). A significant effect of the group and time interaction was recorded, however, indicating that the passing of time influenced the patients differently, depending on whether they had visualized the video or not. Specifically, in the control group, no significant difference was observed in the STAI score between baseline and the end of the biopsy procedure (*p* = 0.542), while in the experimental group the score at the end of the procedure was significantly lower than at baseline (*p* < 0.001). At the end of the operation, the patients in the experimental group presented a significantly lower anxiety state score than the patients in the control group (*p* = 0.012).

## 4. Discussion

The present study found that providing patients with information about the oral biopsy procedure in video format reduces anxiety, with differences being observed in the MDAS and STAI scores at the end of the biopsy procedure versus baseline. No such changes were observed in the control group.

Biopsies continue to be perceived by patients as aggressive and invasive procedures [9,10]. Although biopsy is a simple technique that produces only mild injury, it does have a number of associated adverse effects. Excessive patient fears in relation to interventional procedures of this kind can give rise to behaviors that complicate or can even impede surgery [17,24]. This is a matter of considerable concern, particularly since oral biopsies can prove crucial for diagnosing potentially serious diseases, and such patient fears can cause a delay in establishing the diagnosis [1,2,3,4].

Drug-based interventions, including the administration of anxiolytics and sedative agents, have been used to reduce preoperative anxiety among patients. However, in view of the short duration of the biopsy procedure and the potential side effects of these drugs, non-pharmacological measures can also be considered [30]. One option, in this regard, is the use of multimedia tools that improve patient understanding of the procedure, reduce preoperative anxiety and increase patient satisfaction [10,18,19]. Preoperative anxiety induces transient physical, psychological and behavioral alterations in patients. Based on the existing evidence, a high level of preoperative anxiety can result in difficulties or complications in the postoperative period. The physical consequences usually involve delayed wound healing (secondary to immune suppression) and postoperative recovery, increased postoperative pain, functional impairment and an increased need for anxiety and pain management [30].

The literature shows that providing patients with full and relevant information about the procedure can help them to be prepared and improve their capacity to handle their disease and the side effects. Videos can be used in different ways as an initial information source for obtaining informed consent, or as a method to lessen anxiety [10,31,32]. In this respect, Kesari et al. [33] compared the anxiety levels between patients who watched a video of their own cystoscopy procedure versus those who did not, and reported no significant effects. On the other hand, Tanaka et al. [34] presented patients with a real-time video of their arthroscopy procedure and recorded high postoperative patient satisfaction scores. Likewise, the use of multimedia instruments has been shown to improve patient understanding, reduce anxiety in relation to the procedure and elevate patient satisfaction in different surgical scenarios.

According to Lin et al. [18] and Kinnersley [25], when the professional provides information, three basic factors can influence the level of anxiety experienced by the patient: the amount of information required by the patient; the information which the professional chooses to provide; and the way in which the professional transmits the information.

With regard to the amount of information we offer the patient, there is a lack of consensus as to the scope and conceptual dimensions that should be included [34,35,36,37,38]. Thus, while some authors consider that a greater amount of information will reduce anxiety before surgery, others are of the opinion that this will actually increase patient anxiety [23,24,25]. Vallerand et al. [39] reported that increasing the amount of preparatory information about the postoperative period significantly augments pain relief and patient satisfaction with pain control, with no greater consumption of analgesics.

Another factor that affects the level of patient anxiety is the way in which the information is provided. Research has shown patients to experience difficulties in retaining large amounts of information. Furthermore, patients are often unable to visualize how the biopsy will be carried out [10]. Likewise, some professionals may lack the communication skills needed to explain the information in detail. These problems imply that patients can receive incomplete information. Therefore, the professional should focus on developing more effective ways to convey the information, helping the patients and their relatives to make rational decisions, even in the most demanding scenarios [20].

The way in which the information is provided is indeed important [23,24,25]. The traditional approach has been to provide verbal and/or written information. However, some patients can have problems in understanding the information presented in such ways [10,24]. Some studies have evidenced that the use of videos results in greater patient satisfaction and knowledge about the procedure and its risks [10,20]. Patients informed by means of a video are better able to retain the information, and this strategy has also been used in relation to the removal of impacted third molars [18,19]; however, the use of video-based information among patients requiring an oral biopsy has not been analyzed to date.

Video-delivered information can prove time saving, and transmits many more stimuli than verbal communication alone [10,25,36]. Another aspect that must be considered is patient counseling on the search for information, since personal concerns typically lead patients to explore other data sources (including videos) online, where the global quality of the information is often questionable [39,40].

In the present study we made use of the validated STAI, MDAS and HAD scales. The STAI, which is widely used to assess anxiety, explores transient anxiety state and more stable anxiety trait, and the patients report on how they feel both at the present timepoint and in general. In this regard, we recorded changes in the STAI and MDAS scores among the patients in the experimental group. In contrast, the pre- and post-biopsy hemodynamic parameters (systolic and diastolic blood pressure, heart rate and oximetry) showed no significant changes in either group.

The results obtained show that video-based information offers the added advantage of reproducibility and the capacity to give the professional more time to engage in other activities [10]. In turn, videos adapted to specific patient groups can improve communication efficacy. An important aspect in this regard is to design the video to be culturally sensitive to the target population. In our case, the video was adapted to the population in Guatemala, characterized by a limited educational level or limited health knowledge.

On the other hand, videos also offer the advantage of allowing the patients to repeatedly visualize parts of the recording if necessary. Gagliano et al. [41] found that one of the most effective uses of videos is that the provision of role models increases knowledge, cooperation and coping skills during stress. Practical implications: the present study found that providing patients with information about the oral biopsy procedure in video format reduces anxiety

The present study has some limitations, since we did not take into account the patient preferences with respect to the provision of much or little information. In this regard, some patients might not want to know more details on the risks of the procedure. Another limitation is the fact that we did not include information on previous experiences with local anesthesia and satisfaction with the procedure. Although we decided not to include these aspects in the study, they can prove relevant in future research.

In the present study, the role of the video was simplified, and we focused on presenting the surgical procedure, the factors influencing improvement and the general risk of complications.

## 5. Conclusions

In conclusion, oral biopsy is a stressing event that can generate patient anxiety. The present study is the first randomized controlled trial to compare information on the oral biopsy procedure delivered to the patient in video format versus traditional verbal information. The video was seen to reduce anxiety among the patients. Further studies are needed to identify the subgroups of patients that can benefit more from video-based information.

## Figures and Tables

**Table 1:** Description and comparison of the demographic data and habits of the study subjects.

	Total	Video		*p*-Value
		No	Yes	
**Age**, *mean (SD)*	40.5 (15.3)	42.1 (16.3)	39.0 (14.2)	0.276
**Gender**, *n (%)*				0.847
Female	79 (65.8)	39 (65)	40 (66.7)	
Male	41 (34.2)	21 (35)	20 (33.3)	
**Educational level**, *n (%)*				0.486
Illiterate/primary	46 (38.3)	22 (36.7)	24 (40)	
Secondary	42 (35)	24 (40)	18 (30)	
University	32 (26.7)	14 (23.3)	18 (30)	
**Smoking**, *n (%)*				0.752
No	109 (90.8)	55 (91.7)	54 (90)	
Yes	11 (9.2)	5 (8.3)	6 (10)	
**Alcohol**, *n (%)*				1
No	110 (91.7)	55 (91.7)	55 (91.7)	
Yes	10 (8.3)	5 (8.3)	5 (8.3)	
				
**Bruxism**				0.59
Yes	25 (21.4)	14 (23.3)	11 (19.3)	
No	92 (78.6)	46 (76.7)	46 (80.7)	
				
				
**Oral hygiene (brushing)**				<0.001
No	10 (8.3)	10 (16.7)	0 (0)	
Once a day	23 (19.2)	23 (38.3)	0 (0)	
Twice a day	50 (41.7)	14 (23.3)	36 (60)	
Three times a day	37 (30.8)	13 (21.7)	24 (40)	

**Table 2:** Description and comparison of the oral biopsy parameters.

	Total, *n (%)*	Video, *n (%)*		Test	*p*-Value
		No	Yes		
**Type of biopsy**					<0.001
Incisional	35 (29.2)	27 (45)	8 (13.3)		
Excisional	85 (70.8)	33 (55)	52 (86.7)		
**Anesthetic technique**					0.432
Truncal	17 (14.2)	10 (16.7)	7 (11.7)		
Infiltrating	103 (85.8)	50 (83.3)	53 (88.3)		
**Number of carpules**					0.251
1	78 (65)	42 (70)	36 (60)		
2	42 (35)	18 (30)	24 (40)		
**Sutures**					0.232
0	76 (63.3)	35 (58.3)	41 (68.3)		
1	9 (7.5)	7 (11.7)	2 (3.3)		
2	31 (25.8)	15 (25)	16 (26.7)		
3	4 (3.3)	3 (5)	1 (1.7)		
**Duration of the biopsy**					0.002
0–10 min	4 (3.3)	1 (1.7)	3 (5)		
11–20 min	62 (51.7)	22 (36.7)	40 (66.7)		
21–30 min	40 (33.3)	26 (43.3)	14 (23.3)		
31–40 min	14 (11.7)	11 (18.3)	3 (5)		
**Difficulty of the biopsy**					0.711
Simple	70 (58.3)	36 (60)	34 (56.7)		
Moderately difficult	50 (41.7)	24 (40)	26 (43.3)		

**Table 3:** Description and comparison of the hemodynamic parameters.

	Total, *Mean (SD)*	Video, *Mean (SD)*		Student *t*-Test
		No	Yes	*p*-Value
**Systolic blood pressure (mmHg)**	129.42 (12.7)	130.10 (11.8)	128.73 (13.6)	0.557
**Diastolic blood pressure (mmHg)**	78.82 (5.4)	79.22 (3.3)	78.42 (6.8)	0.416
**Heart rate (bpm)**	79.69 (7.0)	80.15 (9.1)	79.23 (4.0)	0.478
**Oximetry (%)**	97.38 (1.8)	97.48 (2.3)	97.28 (0.9)	0.532

**Table 4:** Means (SD) and statistical contrasts between groups of the hemodynamic parameters.

	Measure		Intra-Subject Effects	
	Baseline, *Mean (SD)*	Final, *Mean (SD)*	Time	Group*Time
			F(df); *p*-Value (η^2^)	F(df); *p*-Value (η^2^)
**Systolic blood pressure (mmHg)**			*F*(1;118) = 0.237; *p* = 0.627 (0.002)	*F*(1;118) = 2.056; *p* = 0.154 (0.008)
No video	130.10 (11.76)	128.07 (9.99)		
Yes video	128.73 (13.60)	129.97 (19.99)		
*Total*	129.42 (12.68)	129.02 (15.76)		
**Diastolic blood pressure (mmHg)**			*F*(1;118) = 0.435; *p* = 0.511 (0.004)	*F*(1;118) = 0.65; *p* = 0.422 (0.005)
No video	79.22 (3.30)	79.13 (3.09)		
Yes video	78.42 (6.84)	79.25 (9.13)		
*Total*	78.82 (5.37)	79.19 (6.79)		
**Heart rate (bpm)**			*F*(1;118) = 1.156; *p* = 0.284 (0.01)	*F*(1;118) = 2.205; *p* = 0.140 (0.009)
No video	80.15 (9.14)	79.08 (6.64)		
Yes video	79.23 (4.01)	79.62 (3.48)		
*Total*	79.69 (7.04)	79.35 (5.29)		
**Oximetry (%)**			*F*(1;118) = 1.615; *p* = 0.206 (0.014)	*F*(1;118) = 0.002; *p* = 0.965 (0)
No video	96.48 (2.32)	96.72 (1.91)		
Yes video	97.28 (0.94)	97.53 (1.28)		
*Total*	96.88 (1.81)	97.13 (1.67)		

df: degrees of freedom. η^2^: partial eta-squared (effect size). *: xxxx, delete

**Table 5:** Means (SD) and statistical contrasts between groups of the scales Corah’s Modified Dental Anxiety Scale (MDAS).

	Measure		Intra-Subject Effects	
	Basal, *Mean (SD)*	Final, *Mean (SD)*	Time	Group Time
			F(df); *p*-Value (η^2^)	F(df); *p*-Value (η^2^)
**MDAS**			*F*(1;118) = 3.991; ***p* = 0.048** (0.034)	*F*(1;118) = 5.122; ***p* = 0.025** (0.042)
No video	8.18 (2.88)	8.27 (2.55)		
Yes video	8.10 (4.48)	7.33 (3.38)		
*Total*	8.14 (3.75)	7.80 (3.02)		
**STAI State**			*F*(1;118) = 9.929; ***p* = 0.002** (0.078)	*F*(1;118) = 16.136; ***p* < 0.001** (0.12)
No video	30.95 (6.09)	31.25 (6.66)		
Yes video	31.23 (4.67)	28.75 (3.55)		
*Total*	31.09 (5.40)	30.00 (5.46)		
**HAD Anxiety**			*F*(1;116) = 0.003; *p* = 0.959 (0)	*F*(1;116) = 1.149; *p* = 0.286 (0.01)
No video	7.98 (2.54)	7.81 (2.00)		
Yes video	7.07 (3.78)	7.25 (3.36)		
*Total*	7.53 (3.24)	7.53 (2.77)		
**HAD Depression**			*F*(1;117) = 2.12; *p* = 0.148 (0.018)	*F*(1;117) = 0.475; *p* = 0.492 (0.004)
No video	7.22 (3.32)	7.35 (2.56)		
Yes video	6.82 (3.32)	5.58 (2.66)		
*Total*	6.22 (3.46)	6.47 (2.75)		

df: degrees of freedom. η^2^: partial eta-squared (effect size).
